# Marchiafava–Bignami Disease Associated with Spinal Involvement

**DOI:** 10.1155/2020/8867383

**Published:** 2020-10-28

**Authors:** Jhon Perea, María Belén Luis, Luciana Grimanesa Lázaro, Sergio Scollo, Agustina Tamargo, José Crespo, Maira Avalle, Horacio Solarz, Nora Fernández Liguori, Ricardo Alonso

**Affiliations:** ^1^Department of Neurology, Sanatorio Güemes—University Hospital, Buenos Aires, Argentina; ^2^Anatomical Pathology, Sanatorio Güemes—University Hospital, Buenos Aires, Argentina

## Abstract

Marchiafava–Bignami disease (MBD) is a rare disorder of unknown etiology, strongly associated with alcoholism and malnutrition. MBD causes primary involvement of the corpus callosum, leading to confusion, dysarthria, seizures, and frequent death. We report the case of a 54-year-old male without a history of alcoholism or known malabsorption disease, who presented with altered consciousness and neurologic impairment. Complex B deficiency was addressed. Magnetic resonance imaging (MRI) showed typical corpus callosum lesions. The clinical features and radiologic images suggested spinal cord involvement. Brain histopathologic findings were consistent with MBD. Despite vitamin replacement therapy, he had a poor outcome.

## 1. Introduction

Marchiafava–Bignami Disease (MBD) is a rare disorder that affects adults between 40 to 60 years of age, with a male predominance, and a history of chronic alcoholism and/or malnutrition. It has been associated with vitamin B complex deficiency, and its main feature is progressive demyelination and necrosis of the corpus callosum [1]. There is also involvement of other cerebral structures, such as hemispheric white matter, middle cerebellar peduncle, and basal ganglia. Few cases of spinal cord involvement were reported [2, 3]. Clinical features are heterogeneous and nonspecific. MBD can cause impairment of consciousness, neuropsychiatric symptoms, dysarthria, tetraparesis, hypertonia, ataxia, seizures, and symptoms of interhemispheric disconnection [4–6]. A clinical and radiologic classification proposed by Henrich divides patients into two types: *type A*, characterized by involvement of the entire corpus callosum, and *type B*, characterized by partial or focal callosal lesions [5, 7, 8]. Although the prognosis is variable, the mortality is high. Early thiamine replacement therapy is associated with a better outcome, whereas the effectiveness of steroids and other treatments remains hypothetical [9, 10]. Different etiologies (infectious, vascular, metabolic, demyelinating, or neoplastic) can cause corpus callosum signal abnormalities. Therefore, the exclusion of differential diagnoses is essential [11, 12]. We present the case of a 54-year-old man suffering from vitamin B complex deficiency and spinal cord involvement, with histopathologic examination consistent with MBD.

## 2. Case Report

**Figure 1:**
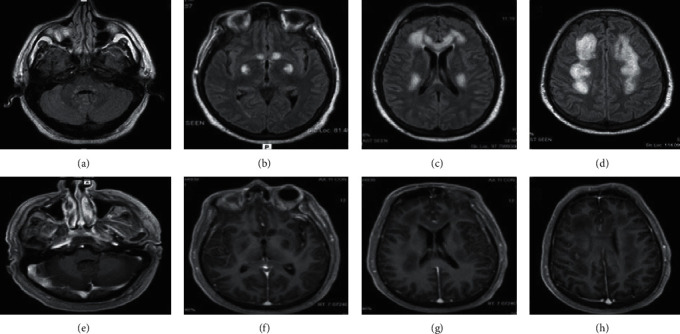
FLAIR brain MRI showing hyperintensity involving peduncles and middle cerebellar peduncles (a). Head of the caudate nucleus and both globus pallidus (b). Corpus callosum and corona radiata (c, d). Gadolinium T1 sequence (e–h) showing focal pathological enhancement in the right parietal lobe (h).

A 53-year-old male was admitted to our hospital due to progressive weakness and impaired state of consciousness. He had a personal history of noninsulin-dependent diabetes, with no history of alcoholism or malnutrition. Two months before admission, he presented with lower limbs weakness, which rapidly progressed leading to severe gait impairment and wheel chair bound. Initial evaluation was performed at another medical center and was referred to our hospital one week later. On admission, he had signs of respiratory distress, which led to intubation and transfer to the intensive care unit. At the initial examination, the patient was in a spontaneous coma. Pupils were symmetric and reactive. There were no signs of cranial nerve impairment, and brainstem reflexes were normal. He had an abnormal extension posture of the upper limbs, with normal withdrawal of lower limbs to painful stimulus. Muscle tone and reflexes were normal, and no meningeal signs were found. A brain MRI showed hyperintensities and extended T2-signal and fluid-attenuated inversion-recovery sequence (FLAIR) involving cerebral peduncles and middle cerebellar peduncles, parietal corpus callosum, and corona radiata (Figures 1(a)–1(d)).

**Figure 2:**
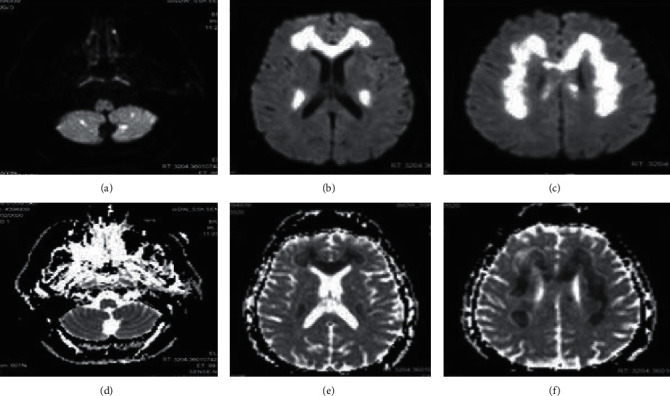
DWI brain MRI showing a hypersignal in the cerebellar hemisphere (a), corpus callosum (b, c), and corona radiata (c). There was a corresponding decrease of ADC values on the ADC map (d–f).

On reviewing of diffusion-weighted imaging signal (DWI), there was homogenous and symmetrical hypersignal throughout the lesion and a corresponding decrease of apparent diffusion coefficient (ADC) values on the ADC maps diffusion restriction (Figures 2(a)–2(f)).

**Figure 3:**
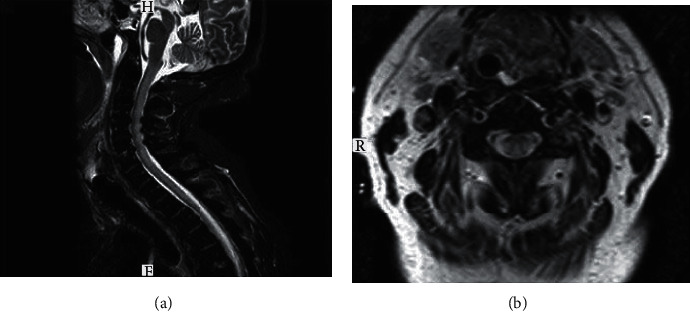
Short-tau inversion-recovery (STIR) sequence and T2: sagittal and axial slice. (a, b) extensive medullar hyperintensity of the lateral and posterior cords at levels C2-T1.

Furthermore, there was an abnormal focal enhancement in corona radiata of the right parietal lobe (Figures 1(e)–1(h)). Spinal cord MRI showed a longitudinally extensive, nonenhancing STIR-hyperintense lesion from C2-T1 with mild edema (Figures 3(a) and 3(b)).

Based on the clinical and image findings, MBD versus central nervous system demyelinating disorders (i.e., aggressive multiple sclerosis, acute disseminated encephalomyelitis, or neuromyelitis optica spectrum disorder) were suspected. Blood count showed severe pancytopenia with megaloblastic anemia. Serum vitamin B1 and B12 levels were very low. Levels of serum homocysteine were elevated, while malonic acid and serum folic acid were normal. CSF analysis revealed hyperproteinorrachia(468 mg/dl) and increased lactic acid (7 mmol/l). Oligoclonal bands, AQP4 IgG and anti-MOG IgG, were negative. Viral and bacterial infections were ruled out (viral PCR screen and bacterial cultures). Other laboratory tests are shown in Table 1.

**Figure 4:**
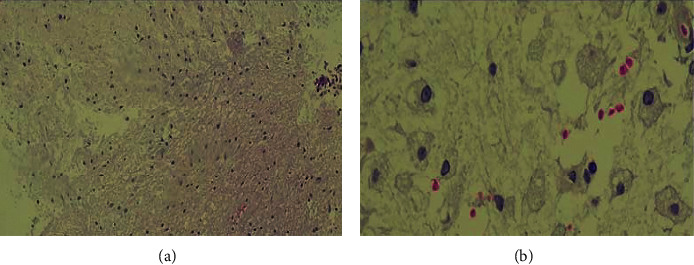
Histopathology of a biopsied subcortical lesion of the right frontal lobe (hematoxylin and eosin staining). (a) The bottom of the image shows normal eosinophilic white matter and demyelinated tissue at the top. (b) Magnification of demyelination zones with macrophage infiltration.

Treatment with IV thiamine: high doses of parenteral B12 vitamin and methylprednisolone pulses (1000 mg/day for 5 days) were started. A brain biopsy was performed at the 20^th^ day of hospitalization, revealing white matter necrosis, areas of demyelination, and gliosis, as well as macrophage infiltrates and perivascular lymphocytes (Figure 4).

These findings were also consistent with MBD. Although laboratory parameters improved with treatment (reaching normal values of both vitamins), there was no clinical improvement, as shown in Table 2. The patient died on day 125 of hospitalization.

## 3. Discussion

Marchiafava and Bignani were Italian pathologists who, in 1903, described a syndrome characterized by selective demyelination and necrosis of the corpus callosum. This entity affected consumers of large volumes of red wine [13]. Later on, this affection was also found in people with severe nutritional deficits [14]. It has been associated with vitamin B complex deficiency, primarily thiamine deficiency. In a few cases, low cyanocobalamin serum levels were also associated with spinal cord involvement [2, 3]. Even though our patient had no personal history of alcoholism or malnutrition, low serum levels of vitamins B1 and B12 were found. The pathophysiological mechanism of MBD remains unclear. Possible mechanisms include cytotoxic edema, the breakdown of the blood-brain barrier, demyelination, and necrosis. Cytotoxic edema is possibly the underlying mechanism involved in the early stages, while demyelination and necrosis take place in later stages. Morel's laminar sclerosis of cerebral cortex is seen [15].

MBD clinical manifestations vary and are nonspecific, posing a diagnostic challenge, and different clinical presentations were described [16]. Acute MBD causes seizures, altered consciousness, and limb hypertonia. Subacute MBD is characterized by confusion, dysarthria, behavioral abnormalities, visual disturbance, memory deficits, signs of interhemispheric disconnection, apraxia, tetraparesis, and gait disorders. The chronic clinical presentation of MBD is the least common and characterized by chronic dementia. Severe impairment of consciousness and neurocognitive deficits at the beginning appear to be indicators of a poor prognosis [1, 5, 15]. Clinical manifestation in our patient was consistent with an acute/subacute presentation. Typical MRI findings in patients with acute MBD include symmetric T2-signal and FLAIR hyperintensity of the corpus callosum, with low signal intensity in T1-signal sequences. DWI restriction of cerebral cortex, hemispheric white matter, middle cerebellar peduncles, and basal ganglia was also described. Contrast enhancement of the corpus callosum, which resolves in the subacute stage, has been reported. In some cases, the T2-signal of the corpus callosum normalizes, evolving into symmetric atrophy [5, 15]. Classifying our patient into *type A or B* was difficult, as he presented with clinic-radiological features of both types. T*ype A* is characterized by severe impairment of consciousness, seizures, dysarthria, and hemiparesis. Imaging findings are swelling of the entire corpus callosum and extra callosal lesions. It has been associated with a poor outcome (death in up to 21% of cases). In contrast, *type B* presents with slight impairment of consciousness, focal callosal lesions on MRI, and a lower mortality rate [5, 17]. An interesting feature of our case was the extensive spinal cord involvement from C2 to T1. We thought that this finding could be associated with B12 hypovitaminosis. The combination of cerebral and spinal cord involvement has only been reported in two previous MBD cases [3, 18]. Histopathological findings of MBD showed demyelination (particularly of corpus callosum central fibers) with myelin breakdown products, foamy macrophages, perivascular lymphocytes, gliosis, and white matter necrosis [19, 20]. Most of the cases reported show postmortem examinations. In our case, a brain biopsy was performed, and the findings were consistent with MBD.

Regarding prognosis, MBD is usually associated with a high mortality, although some cases with a favorable outcome have been reported [9]. In a large published series, out of 250 patients, only 20 (8%) presented a favorable recovery [7]. Alcoholic MBD was associated with a less favorable outcome, and death was caused mainly by infectious complications [1, 21]. Related to vitamin B complex deficiency association, thiamine replacement has become a widespread treatment option, and better outcome was described in subjects treated in the acute stage. Corticosteroids may reduce brain edema, suppress demyelination, stabilize the blood-brain barrier, and reduce inflammation. Although some publications have reported an improvement with this therapy, its benefits are still doubtful and further research is required [12, 21].

## 4. Conclusion

MBD is an uncommon entity, which represents a neurological emergency. It requires strict multidisciplinary care in the acute stage, due to its high morbimortality. Our patient had an atypical presentation with spinal cord involvement without typical comorbidities. His diagnosis was a real clinical challenge. Our hypothesis in this case was a double mechanism of neuronal degeneration due to nutritional deficiency of both vitamins B1 and B12.

## Figures and Tables

**Table 1:** Blood chemical values.

Variable	Value	Reference values
Hematocrit (%)	25	40–50
Hemoglobin (g/dl)	8.8	13.9–17.2
Mean corpuscular volume (*μ*m^3^)	102.4	85–95
	35	27–33
Medium corpuscular hemoglobin (pg)		
White cells (per mm^3^)	3100	4000–12000
Differential count (%)		
Neutrophils	61	50–65
Lymphocytes	30	25–45
Monocytes	3	2–8
Eosinophils	6	0–4
Platelets (per mm^3^)	91000	15000–450000
Glucose (mg/dl)	164	60–110
Urea nitrogen (mg/dl)	54	18–45
Creatinine (mg/dl)	0.53	0.7–1.2
Sodium (mmol/liter)	148	138–150
Potassium (mmol/liter)	3.4	3.6–5
Chloride (mmol/liter)	116	98–105
Coagulogram		
TP	99	70–100
KPTT	30	30–45
Hepatogram		
Total bilirubin	1.2	0.4–1.4
ASAT	19.5	1–31
ALAT	19.8	1–34
Vitamin B1 (*μ*g/l)	3.4	4.0–20
Vitamin B12 (pg/ml)	<50	180–900
Folic acid (ng/ml)	15	>5.3
Homocysteine (mmol/l)	>65	5–15
Malonic acid (nmol/l)	103	<0.4
TSH (mc*μ*i/ml)	0.2	0.27–4.2
Serologies		
HIV	Negative	
Syphilis	Negative	
Hepatitis B	Negative	
Hepatitis C	Negative	
Erythrocyte sedimentation rate, mm	37	1–10
CSF flow cytometry	No immunophenotypic abnormalities were observed	
Oligoclonal bands	Type I	
Anti-MOG antibody IgG	Negative	
Aquaporin 4 IgG	Negative	

**Table 2:** Blood chemical values after treatment.

Variable	Value	Reference values
Hematocrit (%)	35.5	40–50
Hemoglobin (g/dl)	11.1	13.9–17.2
Mean corpuscular volume (*μ*m^3^)	30.4	85–95
	97.3	27–33
Medium corpuscular hemoglobin (pg)		
White cells (per mm^3^)	10100	4000–12000
Differential count (%)		
Neutrophils	90	0–65
Lymphocytes	6	25–45
Monocytes	4	2–8
Eosinophils	0	0–4
Vitamin B1 (*μ*g/l)	15.8	4.0–20
Vitamin B12 (pg/ml)	>2000	180–900
